# The Prognostic Significance of the Preoperative Full Blood Count after Resection of Colorectal Liver Metastases

**DOI:** 10.1155/2009/425065

**Published:** 2009-09-08

**Authors:** K. Dajani, D. A. O'Reilly, N. De Liguori Carino, P. Ghaneh, G. Poston, A. Wu

**Affiliations:** Department of Surgery, University Hospital Aintree, Lower Lane, Liverpool L9 7AL, UK

## Abstract

*Introduction*. Increased preoperative platelet and neutrophil counts are risk factors for decreased survival in several different malignancies. Our aim was to investigate the relationship between overall or disease-free survival after resection of CRLM and the preoperative haematological parameters. *Methods*. We reviewed a cohort of 140 patients who underwent resection of CRLM with curative intent, utilising prospectively maintained databases. Patient demographics, operative details, FBC, CRP, INR, histopathology results, and survival data were examined. Kaplan-Meier survival and Cox regression analyses were used to determine the impact of all variables on survival. *Results*. 140 patients (96 males) with a median age of 67 years (range 33–82 years) underwent resection of CRLM. A significant correlation was exhibited between preoperative platelet count and neutrophil count (rho = 0.186, *P* = .028). When modelled as continuous covariates in a Cox regression hazards, an increased preoperative platelet (*P* = .02) and neutrophil counts (*P* ≤ .001) were significantly associated with overall survival. Of the haematological parameters assessed only preoperative platelet count showed a strong trend of association with disease free survival; however this failed to reach statistical significance (*P* = .076). *Conclusions*. Increased preoperative platelet and neutrophil counts are independent risk factors for decreased survival in patients undergoing resection of CRLM in our series of patients. These findings require validation in larger studies to determine their relationship with survival. Further research into the role of these cell types in tumour progression, particularly in the development and inhibition of angiogenesis, is warranted.

## 1. Introduction

The utility of surgical resection of colorectal liver metastases (CRLM) is clearly established. A large number of substantial prospective and retrospective studies consistently show five-year survival rates following liver resection of 30%–50%, depending on selection criteria [1]. Recently, a shift has occurred in the criteria used for assessing resectability, from morphological criteria to those based on whether a macroscopically and microscopically complete (R0) resection of the liver can be achieved while leaving sufficient functioning liver (the “future liver remnant”) [2–6]. 

This, however, does not give useful information on prognosis following resection. Several studies have identified possible preoperative prognostic factors for survival including stage of primary tumour, preoperative carcinoembryonic antigen (CEA) level, disease free interval, size and number of hepatic metastases, and the presence of extrahepatic disease [7]. A systematic review of these studies has identified a worse prognosis and earlier tumour recurrence associated with resection margin involvement, lymph node metastasis, and the presence of bilobar liver disease [1]. However, little is known about the influence of preoperative haematological factors on prognosis following CRLM resection.

Preoperative haematological parameters and markers of the systemic inflammatory response have been correlated with prognosis in several other malignancies. An increased preoperative platelet count has been identified as an adverse prognostic indicator in bronchial [8], gastric [9], gynaecological malignancies [10, 11], and colorectal cancer [12]. It has also been shown to be an independent prognostic indicator of survival in patients undergoing resections for squamous cell carcinoma of the oesophagus [13] and adeno-carcinoma of the pancreas [14, 15]. Similarly, lymphocyte count has been identified as a prognostic indicator in gastric [16] and breast malignancy [17]. The C-reactive protein (CRP), a marker of local and systemic inflammation, is also related to survival. A raised CRP has been associated with a poorer prognosis in patients with gastro-oesophageal, colorectal cancer, and CRLM [18–20]. A possible explanation lies in the process of tumour angiogenesis, which is a complex interaction between both host and tumour involving pro- and antiangiogenic factors. 

The aim of this study was to investigate the relationship between survival after resection of CRLM and routine preoperative blood tests (FBC, CRP, and INR), whose oncological significance may not be fully appreciated.

## 2. Methods

We reviewed a cohort of 140 patients who had undergone a liver resection for CRLM with curative intent between January 2000 and November 2006 utilising a prospectively maintained database. Additional information was obtained from hospital notes and laboratory reports where required. This patient series represents a single consultant surgeon's consecutive caseload for CRLM resections during this period.

Data on the following variables were obtained: patient demographics, operative details including those of the primary bowel and secondary hepatic resections, tumour stage, histological confirmation of CRLM, margin status, and hepatic nodal status. Initial Dukes stage, perioperative morbidity (e.g., blood loss, transfusion status, length of stay, and complications), preoperative laboratory investigations (haemoglobin, total and differential white cell count, platelet count, INR, and CRP), survival (disease free survival and overall survival), and follow-up data were also recorded. Patients were excluded if no follow-up data beyond hospital admission was available (unless they died during their admission) and if they had undergone a noncurative procedure. Hepatic resection was performed using the Cavitron Ultrasonic Surgical Aspirator (CUSA) and argon plasma coagulation. Intermittent Pringle manoeuvre (of 15 minutes of ischemia followed by 5 minutes of reperfusion) was used only when deemed necessary. Patients were followed-up at HPB specialist clinics. Median follow-up was 21 months (range 1–82 months).

### 2.1. Statistical Analysis

Kaplan-Meier analysis with logrank testing was used for univariate analyses, in order to assess the association between time to death (overall survival), or tumour recurrence (disease free survival), and each of the categorical predictor variables of interest. Correlation between two continuous datasets was analysed with Spearman's rank correlation. The initial univariate analysis was performed using Cox proportional hazards regression for each of the haematological variables of interest. These were modelled as continuous covariates for both univariate and multivariate analyses to more reliably describe their prognostic value [21]. Kaplan-Meier curves were generated to illustrate the survival trends according to the haematological parameters of interest. Variables that showed a trend for association with survival (*P* < .3) were selected for inclusion in the final multivariate Cox proportional hazards model [22]. 

## 3. Results

### 3.1. Patient Demographics and Outcome

Table 1 details the demographic and tumour characteristics of the study population. 140 patients who underwent liver resection for CRLM with curative intent were included in this analysis. The median age was 67 years (range 33 to 82 years). 96 were males. Median disease free interval (time interval between initial colorectal resection and diagnosis of CRLM) is 6.25 months (range 0–64 months). 62 patients had synchronous CRLM. Synchronous tumours are defined as those diagnosed within three months of the initial colorectal resection. Tumour recurrence occurred in 71 patients at local and distant sites during follow-up. 53 patients had recurrence in the liver or in the lungs; the remainder occurred in the pelvis, bones, brain, and nodal recurrence. Median time to recurrence, disease free survival, was 23 months (range of 2–69 months). The median overall survival for the entire cohort was 41 months (range 1–82 months).

The overall 30 days mortality for the entire cohort was 7 patients. Postoperative complications occurred in 23.4% of patients. 118 patients had operative blood loss of <500 mls, 16 patients had blood loss of 500–1000 mL, and 4 patients had >1000 ml of operative blood loss. 9 patients required either intra- or postoperative blood transfusion.

### 3.2. Influence of Preoperative Haematological Factors on Survival

**Figure 1:**
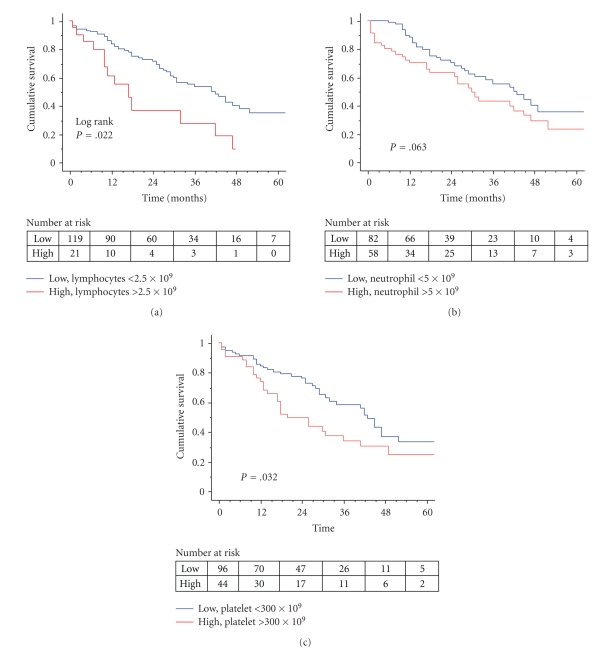
Kaplan-Meier survival curves for illustration of the relationship between the haematological parameters of interest and overall survival. (a) Kaplan-Meier survival curve to illustrate the significant relationship between an increased preoperative lymphocyte count and shorter overall survival. (b) Kaplan-Meier survival curve to illustrate the relationship between an increased preoperative neutrophil count and shorter overall survival. (c) Kaplan-Meier survival curve to illustrate the significant relationship between an increased preoperative platelet count and shorter overall survival.

The distribution and prognostic relevance of preoperative haematological parameters are summarised in Table 2. These factors were analysed as continuous variables for univariate Cox proportional hazards regression. Kaplan-Meier cumulative survival curves to illustrate these relationships are shown in Figure 1. There was significant correlation between preoperative platelet and neutrophil count (Spearman, rho = 0.186, *P* = .028).

Platelet count (*P* = .013), lymphocyte count (*P* = .022), neutrophil count (*P* < .001), CRP (*P* = .011), and INR (*P* = .042) were all significantly associated with overall survival on univariate analysis. However, only the platelet count (*P* = .048), neutrophil count (*P* = .046), and CRP (*P* = .016) demonstrated a significant association with disease-free survival. CRP data were only available for 35 of the 140 patients in this study. Two patients with a CRP > 250 were excluded from the univariate CRP analysis as these represented patients with an underlying infective process. Data on preoperative CEA were only available for 58 of the 140 patients in the study. No significant association between CEA and survival was observed on univariate analysis when modelling CEA as a continuous prognostic variable (Cox, *P* = .954). However, when CEA data were dichotomised into groups (using a cutoff value of 10 ng/mL) the group with an elevated CEA exhibited a trend towards poorer survival (log rank, *P* = .049). Because of the incomplete data for both preoperative CEA and CRP, neither of these factors were included in the subsequent multivariate model.

When analysed in a univariate model, disease-free interval did not exhibit any significant relationships with either overall or disease-free survival following resections of CRLM (HR = 1.000 (95% CI 0.996–1.003); *P* = .853, HR = 0.997 (95% CI 0.993–1.003); *P* = .177, resp.). The presence of a synchronous CRLM also did not exhibit any significant relationship with either disease free or overall survival (*P* = .983, .936, resp.) on univariate analysis.

Median survival times according to histopathological tumour characteristics are shown in Table 3. Only resection margin involvement exhibited a significant relationship with overall survival. However, both nodal involvement and bilobar disease demonstrated a clear trend towards less favourable overall survival. Bilobar involvement and margin status also both demonstrated a significant association with disease-free survival.

Table 4 demonstrates the results of a multivariate analysis including both the haematological and histopathological parameters of interest. These results indicate that both the preoperative platelet and neutrophil count as well as resection margin involvement and hepatic lymph node involvement continue to demonstrate a significant association with overall survival. Resection margin involvement and bilobar disease also proved to be significant predictors of disease free survival.

## 4. Discussion

### 4.1. CRLM and Prognostic Markers of Survival

Prognostic factors commonly reported in the setting of resected CRLM include stage of the primary tumour, preoperative CEA level, disease free interval, size and number of hepatic metastases, the presence of extrahepatic disease, resection margin involvement, lymph node invasion, and the presence of bilobar liver disease [23, 24]. It is clear that these crude morphologic and chronologic factors are inadequate, and further prognostic indicators that reflect tumour biology and the interaction between the tumour and its host are required, not only to help with prognostic information, but for a role in identifying possible therapeutic targets in future research studies.

### 4.2. The Blood Count as a Prognostic Factor in Malignancy

Numerous investigations have reported a relationship between increased preoperative blood platelet count and a poor prognosis in patients with malignant tumours, including gastric cancer, oesophageal cancer, gynaecological malignancies, and bronchial cancers [8–13]. Brown et al. have reported preoperative platelet count to be an independent predictor of prognosis following resections for adenocarcinoma of the pancreas [14]. Similar relationships have been identified in gastric and primary colorectal cancer [9, 12].

Less is known about the association of preoperative peripheral blood leucocyte subsets and prognosis in patients with malignant disease. A study on metastatic gastric cancer identified that pretreatment absolute granulocyte count of <6000/mm^3^, lymphocyte count of >1500/mm^3^, and monocyte count of 3000–9000/mm^3^ were independent predictors of poor prognosis [16]. A high preoperative lymphocyte count carries a worse prognosis in those with breast cancer [17] but was associated with a good prognosis in epidermoid carcinoma of the head and neck [25]. A preoperative monocyte count >300/mm^3^ was found to be a predictor of poor prognosis following liver resections for hepatocellular carcinoma [23] and CRLM [24]. However, a relationship was not identified with Lymphocyte count.

C-Reactive Protein (CRP), a marker of the systemic inflammatory response, was shown to be inversely correlated to prognosis in patients with malignant disease and has been identified as an independent predictor of poor prognosis in those undergoing resections for CRLM [20]. The same relationship has been identified in patients with oesophageal cancer [19] and primary colorectal cancer [18]. International Normalised Ratio (INR), a measure of the extrinsic pathway of coagulation, is related to the concentration of VEGF in patients who have had surgical cytoreduction for advanced ovarian cancer [26]. 

In this study, we have attempted to place the relatively easily measurable haematological parameters as predictive markers within the context of other known blood borne predictive markers of outcome (CEA and CRP), following resection of colorectal liver metastases. Our study has identified a high preoperative platelet count and neutrophil counts and has identified resection margin involvement and hepatic lymph node involvement to be independent predictors of a poorer overall prognosis for patients in this study group. This data also suggests that high preoperative CRP level may hold an inverse relationship to both overall and disease free survival; however, as the data was incomplete, inclusion in the final multivariate model was not possible. Of the variables analysed, only resection margin involvement and bilobar liver disease were independent predictors of tumour recurrence (disease free survival) in those undergoing liver resection of CRLM for patients in this study group; however, an elevated preoperative platelet count exhibited strong relationship with a shorter disease free survival but this failed to reach statistical significance.

An elevated preoperative lymphocyte count and INR exhibited a significant univariate relationship with decreased overall survival in patients undergoing resections of CRLM in our series; however, these parameters did not prove to be independent predictors of prognosis as they failed to reach significance on multivariate analysis. 

### 4.3. The Link between the Blood Count and Angiogenesis

An explanation for our findings may lie in the process of tumour angiogenesis [27]. It is now widely believed that primary tumours and metastatic lesions cannot grow beyond 2 to 3 mm in size in the absence of vascularisation [26] regardless if this is secured by occasional “cooption” of pre-existing capillaries [28] or by active angiogenesis [29]. The role of tumour angiogenesis is not only to ensure continual metabolic and oxygen exchange but also to act as an important source of paracrine stimulation [30] which is achieved through endothelial cell-derived extracellular matrix (ECM), proteases, and cytokines which regulate tumour growth, survival, even invasion, and metastasis [30]. The onset of tumour angiogenesis, angiogenic switch, is the result of a shift in balance between pro- and antiangiogenic factors secreted not only by tumour cells but also by several activated host stromal cells [31, 32]. Among these are mast cells [33], resident macrophages [34], blood-borne mononuclear leukocytes [35], and platelets [36, 37].

Vascular Endothelial Growth Factor (VEGF), a potent angiogenic peptide with biologic effects that include regulation of haematopoietic stem cell development, extracellular matrix remodelling, and inflammatory cytokine regeneration [38], is secreted locally by cancer cells and by inflammatory cells in the blood, including platelets and leucocytes [39–41]. Both VEGF and Platelet Derived Endothelial Cell Growth Factor (PD-ECGF) are related to the vascular density and hence the angiogenic process in solid tumours [42, 43]. VEGF is a potent mitogen for endothelial cells and also increases vascular permeability. PD-ECGF is a powerful chemotaxic agent for endothelial cells in vivo. Both are powerful inducers of angiogenesis and may act synergistically [42, 43].

High levels of VEGF and PD-ECGF are found in serum, platelets, and leucocytes of patients with malignant disease [37], when compared to those of normal individuals [40]. VEGF levels are even higher in disseminated disease [41], and elevated serum VEGF levels are an independent prognostic factor in patients with colorectal cancer [43]. It is postulated that the VEGF in the bloodstream is transported by blood cells, including leucocytes and platelets [40–43]. The cells of cancer patients contain greatly elevated amounts of this major angiogenic growth factor, and this reservoir of VEGF may have a role in tumour angiogenesis and metastases formation.

An alternative explanation for our findings is through the link between chronic inflammation and cancer development. Clinical and experimental data now clearly indicate that persisting chronic inflammation significantly contributes to cancer development [44], and it is increasingly appreciated that persistent humoral immune responses exacerbate recruitment and activation of innate immune cells in neoplastic microenvironments where they regulate tissue remodelling, proangiogenic and prosurvival pathways that together potentiate cancer development [45]. 

As most of the haematological parameters analysed in this study exhibited a strong relation with overall survival, but not with disease free survival one can question whether these markers truly represent underlying tumour biology or if they are purely markers of overall health of the patient undergoing a major resection. As discussed earlier our study has provided some interesting observations which will require validation in larger series of patients before a firm conclusion on their role in prognostication can be reached.

In the not too distant future, understanding tumour biology will probably yield more effective predictive data than the present reliance on morphologic description [23, 24]. Cetuximab, a monoclonal antibody targeted against the EGF receptor which acts to inhibit the k-ras pathway, shows promise as agents to downstage CRLM preoperatively in unresectable lesions [46].

In summary, our study has identified that an increased preoperative platelet count and neutrophil count are independent predictors of a worse prognosis in our study population of patients undergoing hepatic resections for CRLM. These findings merit validation in a larger patient series alongside CEA and CA19-9 as additional prognostic covariates which could not be adequately analysed in the present study.

## Figures and Tables

**Table 1:** Demographic and tumour characteristics from resected CRLM.

Number of patients analysed	140
Median age	67 years
(range)	(33–82)
Male : Female	96 : 44
Synchronous : Metachronous	62 : 78
Unilobar : Bilobar disease	85 : 55
Resection margin	
** *Clear : not clear*	132 : 8
Hepatic lymph node	
** *Involved : not involved*	132 : 8
Recurrence following hepatic resection	
** *Yes : no*	71 : 69
Adjuvant chemotherapy	
** *Yes : no*	47 : 55
** *Unknown*	38
Dukes stage	
** *A : B : C : D*	8 : 45 : 85 : 2

**Table 2:** Univariate survival analysis of preoperative haematological parameters as prognostic covariates in resected CRLM (Cox proportional hazards). (NB hazards ratio for continuous data reflects increase in relative risk of event with each incremental increase in covariate value of 1 unit).

		Overall survival		Disease free survival	
	Median (range)	Hazards ratio (95% CI)	*P*	Hazards ratio (95% CI)	*P*
Platelet count	264	1.004	**.013**	1.003	**.048**
(×10^9^/L)	(129–576)	(1.001–1.006)		(1.000–1.005)	
Lymphocyte count	1.6	1.438	**.022**	1.336	.086
(×10^9^/L)	(0.5–5.0)	(1.058–2.078)		(0.960–1.861)	
Neutrophil count	4.7	1.042	<**.001**	1.019	**.046**
(×10^9^/L)	(2–15)	(1.031–1.054)		(1.006–1.038)	
Haemoglobin	13.6	0.964	.648	0.847	**.022**
(g/dL)	(8.8–18)	(0.824–1.128)		(0.735–0.976)	
INR	1.0	14.334	**.042**	1.130	.930
	(0.8–1.4)	(1.104–186.125)		(0.076–16.822)	
CRP*	7	1.019	**.011**	1.018	**.016**
	(5–128)	(1.004–1.032)		(1.003–1.032)	
CEA**	14.9	1.000	.954	1.001	.300
	(0.0–1337)	(0.999–1.001)		(0.999–1.002)	

*Preoperative CRP data available for 33 patients only. **Preoperative CEA data available for 58 patients only.

**Table 3:** Univariate survival analysis of tumour characteristics as prognostic covariates in resected CRLM.

	Overall survival		Disease free survival	
	Median survival (Months)	*P*	Median survival (Months)	*P*
Dukes stage				
** *A + * B*	45	.241	28	.135
** *C + * D*	31		19	
Unilobar versus bilobar	41	.143	30	**.002**
disease	31		17	
Resection margin				
** *Clear*	41	**.001**	24	<**.001**
** *Not clear *	12		8	
Hepatic lymph nodes				
** *Involved*	42	.067	23	.809
** *Not involved*	26		21	

*Due to the small number of patients with Dukes *A* and *D* tumours (*n* = 7 and *n* = 2, resp.), those with Dukes *A* and *B* were grouped together for analysis and survival times compared with Dukes *C* and *D*.

**Table 4:** Multivariate survival analysis of prognostic covariates in resected CRLM (Cox proportional hazards). (NB hazards ratio for continuous data reflects increase in relative risk of event with each incremental increase in covariate value of 1 unit).

	Overall survival		Disease free survival	
	Hazards ratio	*P*	Hazards ratio	*P*
	(95% CI)		(95% CI)	
Dukes stage	1.115 (0.652–1.908)	.691	1.170 (0.693–1.973)	.557
Resection margin	4.008 (1.567–10.252)	**.004**	6.599 (2.432–17.903)	**.002**
Hepatic lymph nodes	2.709 (1.194–6.145)	**.017**	1.732 (0.736–4.076)	.208
Unilobar versus bilobar disease	1.452 (0.869–2.427)	.154	1.968 (1.211–3.200)	**.006**
Platelet count* (×10^9^/L)	1.004 (1.002–1.007)	**.002**	1.003 (1.000–1.005)	.076
Lymphocyte count* (×10^9^/L)	1.022 (0.731–1.429)	.898	1.183 (0.809–1.731)	.386
Neutrophil count* (×10^9^/L)	1.043 (1.027–1.058)	<**.001**	1.018 (0.995–1.041)	.118
INR*	20.331 (0.869–475.645)	.061	1.813 (0.084–39.233)	.609

*Factors included as continuous covariates for Cox regression.
